# Case report: Fatal hemoptysis after effective treatment with tislelizumab and anlotinib in pulmonary sarcomatoid carcinoma

**DOI:** 10.3389/fonc.2024.1445358

**Published:** 2024-09-30

**Authors:** Chen-Wei Pu, Yong-Fen Ma, Jing-Jing Peng, Zhen-Zhen Wang

**Affiliations:** ^1^ Department of Pulmonary and Critical Care Medicine, Zibo Central Hospital, Zibo, China; ^2^ Department of Gastroenterology, Zibo Central Hospital, Zibo, China; ^3^ Department of Stomatology, Zichuan Economic Development Zone Health Center, Zibo, China; ^4^ Department of Infectious Diseases, Zibo Central Hospital, Zibo, China

**Keywords:** tislelizumab, anlotinib, pulmonary sarcomatoid carcinoma, hemoptysis, adverse effects

## Abstract

Pulmonary sarcomatoid carcinoma (PSC), a rare non-small cell lung cancer (NSCLC) subtype, poses diagnostic and treatment difficulties. Current research explores targeted therapies and immunotherapy to improve patient outcomes. This case report details a male patient diagnosed with PSC via pathology. Tests revealed high levels of PD-L1, a marker suggesting potential benefit from immune checkpoint inhibitors. However, despite bronchoscopic intervention, his advanced stage IIIB cancer (cT3N2bM0) progressed quickly, with progression-free survival (PFS) under 3 months. Following progression, the patient received tislelizumab (anti-PD-1 antibody) and anlotinib (an anti-angiogenic drug) as second-line therapy. This combination showed promise, achieving near-partial remission after the first cycle. Subsequent scans documented continued tumor shrinkage until the patient experienced fatal hemoptysis. This case highlights the potential benefits of combining tislelizumab with anlotinib for PSC. However, it also represents the first reported case of fatal hemoptysis with this specific treatment regimen. This finding emphasizes the need for increased awareness of this potential complication, especially in patients with centrally located PSC treated with anti-angiogenic agents like anlotinib.

## Introduction

Pulmonary sarcomatoid carcinoma (PSC) is a rare and heterogeneous malignancy with diverse histologic features. The 2021 WHO Classification of Thoracic Tumors revised the histological classification of PSC by categorizing pleomorphic carcinoma, pulmonary blastoma, and carcinosarcoma as three distinct entities, while pleomorphic carcinoma itself is further divided into three subtypes: giant cell carcinoma, spindle cell carcinoma, and pleomorphic carcinoma (1). Current understanding suggests these tumors arise from the transformation of the bronchial epithelium through a process known as epithelial-mesenchymal transition (EMT) (2). This aggressive form of lung cancer often exhibits resistance to traditional therapies like chemoradiotherapy (3). Radical surgery offers the best chance of cure, and surgery plus neoadjuvant or adjuvant therapy were significant prognostic parameters (4, 5). A retrospective cohort study showed that adjuvant chemotherapy was associated with improved 5-year overall survival for stage II and III disease (6), but recurrence after surgery is common (7). Unfortunately, treatment options for advanced or recurrent PSC are limited.

Due to its limited prevalence and diverse histological presentations, conducting trials specifically focused on PSC is difficult. Consequently, a substantial portion of the current literature relies on individual case reports, and prospective data remains scarce. The exploration of combination therapies involving immunotherapy and targeted agents is an emerging area of investigation, with even fewer documented cases. This case report describes the clinical course of a patient with refractory PSC treated with a combination of tislelizumab and anlotinib. Despite achieving a promising initial tumor response, the patient unfortunately succumbed to hemoptysis.

## Case report

**Figure 1:**
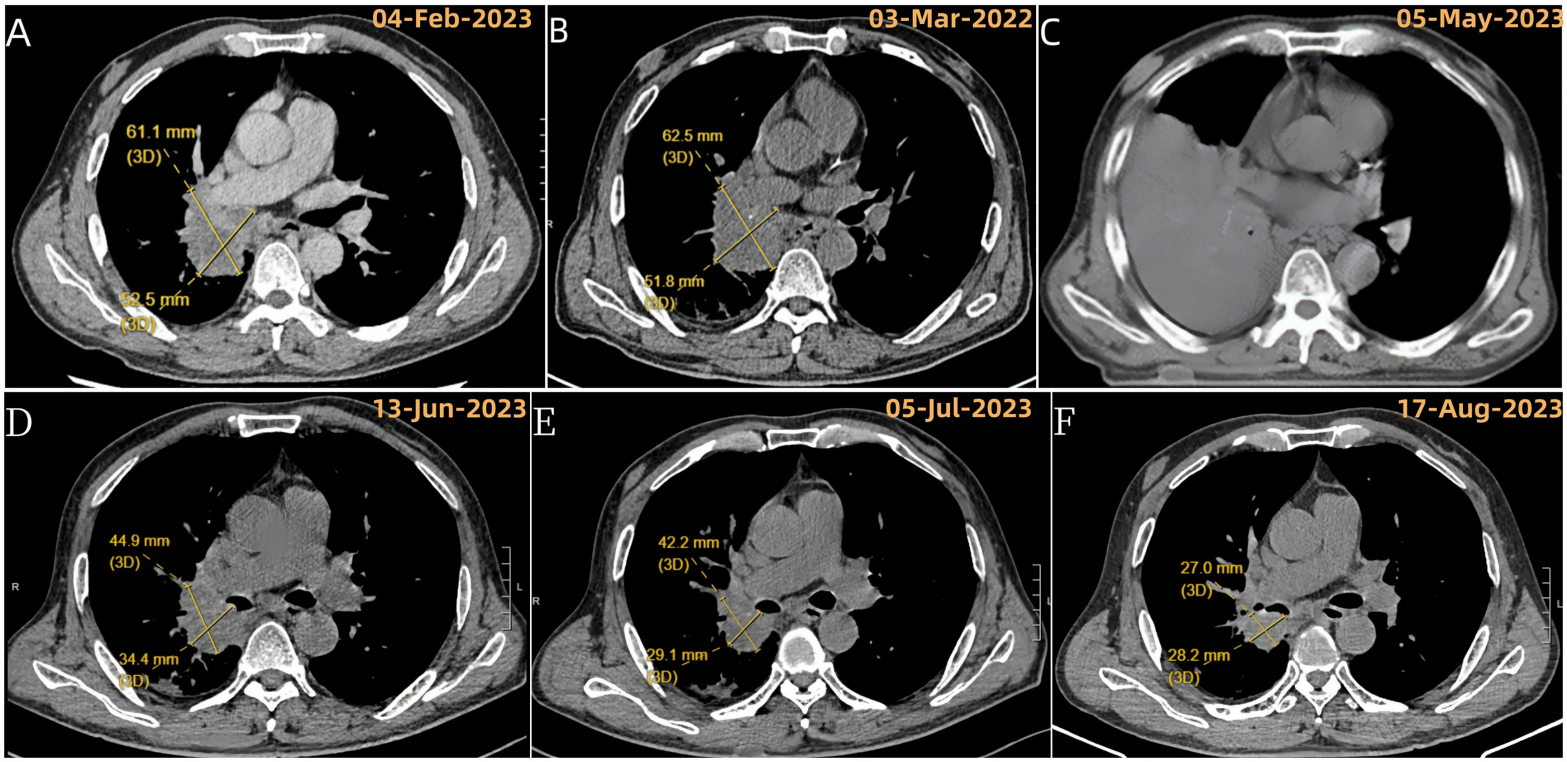
Evolution of right lung target lesion on chest CT. **(A)** Chest enhanced CT at initial presentation (baseline). **(B)** After 2 cycles of chemotherapy; **(C)** After the last tumor resection, before starting second-line treatment (tislelizumab + anlotinib) due to recurrence of atelectasis and inability to measure the target lesion; **(D)** Following the first cycle of tislelizumab + anlotinib; **(E)** After 2 cycles of tislelizumab + anlotinib; **(F)** After 4 cycles of tislelizumab + anlotinib.

**Figure 2:**
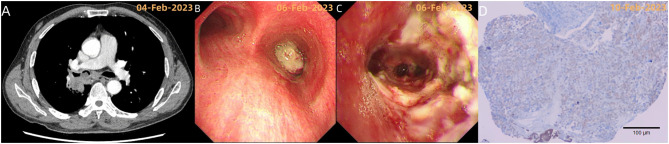
**(A, B)** Chest CT and bronchoscopy reveal a neoplasm obstructing the lumen of the right main bronchus; **(C)** Post-tumor resection bronchoscopy shows the exposure of the openings of the right middle lobe and the basal and dorsal segments of the right lower lobe, indicating the tumor primarily invaded the lumen from the right intermediate bronchus. Bronchoscopic diagnosis: mixed-type malignant central airway stenosis (V zone, VI zone) with a stenosis degree of grade 5 (91-100%); **(D)** Immunohistochemistry showed: tumor cells CKAE1/AE3 (+), CK8/18 (+), CK7 (+), TTF-1 (+), Vimentin (+).

A 59-year-old male patient presented with a 20-day history of chest tightness upon exertion. A chest CT scan with contrast enhancement revealed a right hilar mass measuring approximately 6.11 x 5.25 cm (**Figure 1A**). Additional findings included a neoplasm obstructing the lumen of the right main bronchus (**Figure 2A**), as well as partial right middle lobe volume loss and obstructive atelectasis in the right lower lobe.

### Treatment course

1. February 6, 2023: Bronchoscopic biopsy and initial tumor resection were performed (**Figures 2B, C**).
2. Pathology (**Figure 2D**): Microscopic examination identified a malignant neoplasm, and immunohistochemical analysis confirmed sarcomatoid carcinoma with focal necrosis. Additionally, programmed death-ligand 1 (PD-L1) expression was strong (TPS: 90.0%).
3. Genetic testing of the paraffin-embedded tissue specimen revealed mutations in two genes: TP53 (NM_000546: exon 5: c.569G>T: p.V157F; VAF: 4.80%) and RET (NM_020975: exon 13: c.2296_2297delins2: p.P766K; VAF: 3.60%).

### Evaluation and initial management

Extensive scans, including lymph node ultrasound, abdominal ultrasound, brain MRI with contrast, and bone scan, did not detect any distant spread of the cancer. Based on these findings, the patient was diagnosed with stage IIIB sarcomatoid carcinoma of the right lung (cT3N2bM0). His KPS score of 80 indicated a good ability to perform daily activities. Unfortunately, surgery wasn’t an option due to the tumor’s location near major airways.

The patient received two cycles of chemotherapy (cisplatin and paclitaxel) starting February 17, 2023. A follow-up CT scan on March 31, 2023 (**Figure 1B**), showed stable disease (SD) according to RECIST 1.1 criteria (no significant tumor growth).

### Treatment progression and symptom

On April 1st, 2023, the patient received his third round of chemotherapy and another bronchoscopy to remove some of the tumor. He also began chest radiation therapy.

**Figure 3:**
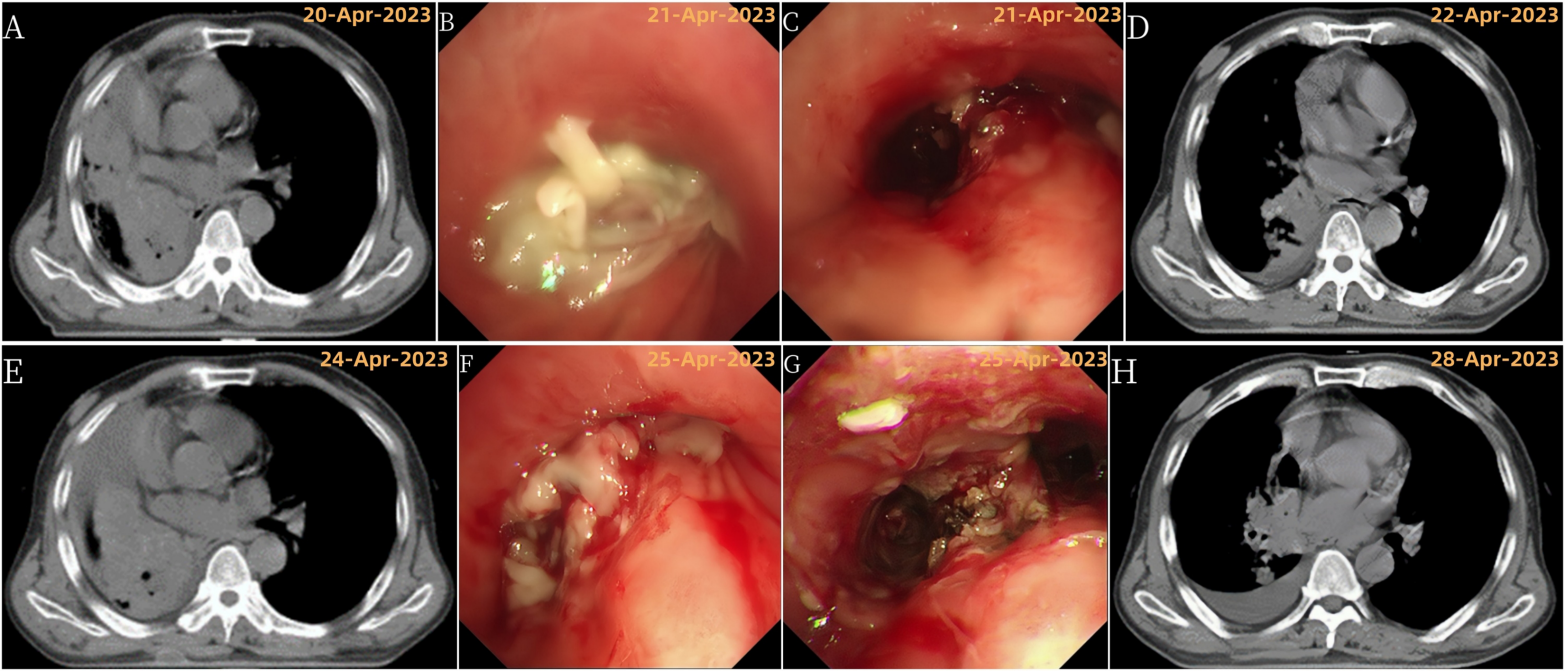
Disease progression and treatment outcomes with multiple bronchoscopic interventions. **(A)** Chest CT showing right total atelectasis and mediastinal shift to the right; **(B)** Bronchoscopy showing complete obstruction of the right main bronchus lumen by a new growth; **(C)** Bronchoscopy after tumor resection showing tumor invasion of the right upper lobe; **(D)** Chest CT showing improvement in right atelectasis and mediastinal return to normal position after tumor resection; **(E)** Chest CT showing recurrence of right total atelectasis and right mediastinal displacement; **(F)** Bronchoscopy showing incomplete obstruction of the right main bronchus, right upper lobe bronchus by a new growth, and occlusion of the right middle bronchus; **(G)** Bronchoscopy after the third tumor resection; **(H)** Chest CT showing improvement in right atelectasis and mediastinal return to normal position after tumor resection.

However, by April 20th, the patient developed wheezing, sweating, and a CT scan showed his right lung had completely collapsed (atelectasis) and shifted the center of his chest (mediastinum) to the right (**Figure 3A**). This indicated his cancer had progressed (PD) according to RECIST criteria.

Despite further bronchoscopies, the lung collapse and mediastinal shift persisted on scans (**Figures 1C**, **3**). The patient also experienced worsening shortness of breath (asthma) and high fever. As a result, his KPS score dropped to 20, signifying a major decline in his ability to perform daily activities. Due to these symptoms, chest radiation therapy was stopped after only 10 sessions.

### Resistance to first-line therapy and initiation of second-line regimen

Despite adding bronchoscopies to remove some of the tumor, the initial chemo-radiation therapy was not effective (failed to achieve disease control). After careful evaluation, the patient began a new treatment regimen on May 9th, 2023. This second-line therapy combined tislelizumab and anlotinib.

The patient completed six cycles of this treatment, ending on September 8th, 2023. Chest scans throughout this period (June, July, August) showed a significant decrease in the tumor size and improvement in the collapsed lung (atelectasis) (**Figures 1D–F**). Additionally, the patient’s ability to perform daily activities (KPS score) improved significantly back to a good level (80). Based on these positive changes, the tumor response was classified as a partial remission (PR) according to RECIST criteria.

### Fatal hemoptysis

Tragically, on September 15, 2023, while eating dinner, the patient experienced a sudden episode of massive hemoptysis (coughing up large amounts of blood). Emergency medical services arrived promptly, but upon arrival, the patient was already in cardiopulmonary arrest. Despite resuscitation efforts, the patient unfortunately succumbed to suffocation secondary to the hemoptysis.

## Discussion

Recent advancements in gene sequencing technology including next-generation sequencing (NGS) have shed light on the similarities between PSC and traditional NSCLC (8). These shared characteristics, including a common origin in epithelial cells and similar immune profiles, suggest targeted therapy might benefit PSC patients who don’t respond well to standard treatments.

While the effectiveness of sevotinib for PSC patients with specific MET gene mutations is well-established, targeted therapies for other mutations like EGFR, ALK, BRAF V600E, and RET mutations rely mainly on case report evidence (9, 10).

This case report describes a patient with PSC harboring two previously unreported mutations: TP53 V157F and RET P766K. The TP53 V157F mutation is a known “lung-enriched mutation” occasionally found in lung adenocarcinoma and squamous cell carcinoma (11). Importantly, neither the TP53 V157F nor the RET P766K mutation has been documented in PSC patients before, and there are currently no available targeted drugs specifically designed for these mutations.

### The PD-1/PD-L1 pathway and rationale for immunotherapy in PSC

The PD-1/PD-L1 pathway plays a critical role in maintaining immune homeostasis by preventing excessive inflammation and autoimmune responses (12, 13). However, tumor cells can exploit this pathway to evade immune attack through the interaction between PD-1 and PD-L1 (14). Immune checkpoint inhibitors (ICIs) targeting the PD-1/PD-L1 pathway, such as anti-PD-1 antibodies, can restore the anti-tumor activity of T cells by blocking this interaction.

PSC demonstrates higher PD-L1 expression compared to other NSCLC subtypes (8, 15). This case report highlights this observation with a tumor proportion score (TPS) of 90% for PD-L1 expression. Emerging evidence suggests the potential efficacy of anti-PD-1 antibodies in treating advanced or metastatic PSC (16, 17).

### Challenges of immunotherapy and rationale for combination therapy

Despite the promise of ICIs, the PD-1/PD-L1 pathway is not the sole mechanism by which tumors evade immune surveillance (18). Dysfunctional blood vessel formation (angiogenesis) within the tumor microenvironment (TME) can further suppress anti-tumor immunity (19). PSC exhibits a propensity for immune evasion independent of the PD-1/PD-L1 pathway and increased vascular infiltration (15, 20). These characteristics suggest a higher likelihood of resistance to ICI therapy in PSC patients. Combining ICIs with anti-angiogenic drugs presents a promising strategy to improve treatment response rates and duration (21).

### Rationale for the treatment regimen

Tislelizumab, an anti-PD-1 IgG4 antibody, is specifically engineered to minimize Fcγ receptor-mediated macrophage binding, thereby reducing T cell depletion through antibody-dependent cellular phagocytosis (ADCP) (22). It boasts a higher affinity for PD-1, a slower dissociation rate, and potentially enhanced anti-tumor activity (22).

Anlotinib is a novel, orally administered tyrosine kinase inhibitor with a broad-spectrum inhibitory effect on tumor angiogenesis and growth (23). It additionally enhances the efficacy of anti-PD-1 immunotherapy by promoting tumor vascular normalization and reversing the immunosuppressive TME (24). At the same time, effector T cells, revitalized following anti-PD-1 immunotherapy, reinforce tumor vascular normalization by enhancing IFN-γ and other pathways, which extends the therapeutic window of anlotinib’s vascular normalization properties (24). Consequently, a positive feedback loop is established, potentially amplifying the anti-tumor effects of both drugs.

A recent study by Qian et al. (17) demonstrated promising results with the combination of tislelizumab and anlotinib in PSC patients. Notably, two patients achieved PR, and four achieved SD. Building on this emerging evidence, the patient in this case report received this same combination (“tislelizumab + anlotinib”) as second-line therapy. Following the first cycle of treatment, chest scans revealed a significant decrease in tumor size and a substantial improvement in the patient’s ability to perform daily activities (KPS score). This indicated a near-partial response based on standard evaluation criteria. Subsequent chest scans confirmed continued tumor shrinkage, solidifying the PR classification.

ICIs can cause adverse effects, with checkpoint inhibitor pneumonitis (CIP) being the most common pulmonary toxicity, especially in patients with NSCLC (25). It’s a serious adverse effect, sometimes fatal, that typically occurs early in treatment with anti-PD-1 antibodies (26). A case reported by Facchinetti et al. linked rapid tumor shrinkage from immunotherapy to fatal hemoptysis in a patient with central lung cancer (27). This case highlights a potential risk but differs from the current case, where the patient received tislelizumab for several cycles with significant tumor shrinkage before experiencing hemoptysis. Combining anti-PD-1 antibodies with anlotinib appears safe in solid tumors like NSCLC, with no increased adverse effects compared to using either medication alone (28, 29). However, anlotinib, an anti-angiogenic drug, can weaken blood vessels and hinder wound healing by affecting blood vessel formation, platelet function, and blood clotting, especially in tissues that rely heavily on a protein called VEGF, like the airway lining (30, 31), as supported by studies showing anlotinib increases hemoptysis risk (32). In this case, the patient developed hemoptysis after anlotinib treatment. While the drug’s information and literature suggest a potential link, the absence of a definitive cause is due to no autopsy being performed. Additionally, the patient’s death prevents further testing. While ongoing CT scans showed tumor improvement, other potential causes of hemoptysis, like bleeding directly from the tumor, cannot be completely ruled out. Therefore, the hemoptysis is classified as “possibly related” to anlotinib with a score of 2 on the Naranjo scale. But this case highlights the importance of heightened awareness of hemoptysis risk, especially for patients with centrally located PSC treated with anlotinib. Hemoptysis risk should be continuously monitored throughout treatment. Interestingly, some studies suggest high-dose anti-angiogenic agents may worsen oxygen and acidosis within the tumor, potentially harming immune cells (33). Conversely, lower doses of anlotinib may normalize tumor blood vessels, potentially improving the safety and effectiveness of anti-PD-1 immunotherapy (34). This suggests a need for large-scale clinical trials to optimize anlotinib dosage in combination with ICIs.

In a word, this study reinforces the promising anti-tumor efficacy of combining anti-PD-1 antibodies with anlotinib for treating PSC patients. However, this case also underscores the importance of careful patient selection and risk assessment for anti-angiogenic therapy. Future research efforts should focus on optimizing combination therapies for PSC, with an emphasis on developing regimens that are both efficacious and have minimal toxicity profiles.

## Data Availability

The original contributions presented in the study are included in the article/supplementary material. Further inquiries can be directed to the corresponding author.
